# Structural basis of specific inhibition of extracellular activation of pro- or latent myostatin by the monoclonal antibody SRK-015

**DOI:** 10.1074/jbc.RA119.012293

**Published:** 2020-02-19

**Authors:** Kevin B. Dagbay, Erin Treece, Frederick C. Streich, Justin W. Jackson, Ryan R. Faucette, Anastasia Nikiforov, Susan C. Lin, Chris J. Boston, Samantha B. Nicholls, Allan D. Capili, Gregory J. Carven

**Affiliations:** Scholar Rock Inc., Cambridge, Massachusetts 02139

**Keywords:** myostatin, transforming growth factor β (TGF-β), muscle atrophy, skeletal muscle, X-ray crystallography, hydrogen exchange mass spectrometry, monoclonal antibody, inhibition mechanism, cell signaling, proteolysis, growth differentiation factor 8 (GDF8)

## Abstract

Myostatin (or growth/differentiation factor 8 (GDF8)) is a member of the transforming growth factor β superfamily of growth factors and negatively regulates skeletal muscle growth. Its dysregulation is implicated in muscle wasting diseases. SRK-015 is a clinical-stage mAb that prevents extracellular proteolytic activation of pro- and latent myostatin. Here we used integrated structural and biochemical approaches to elucidate the molecular mechanism of antibody-mediated neutralization of pro-myostatin activation. The crystal structure of pro-myostatin in complex with 29H4-16 Fab, a high-affinity variant of SRK-015, at 2.79 Å resolution revealed that the antibody binds to a conformational epitope in the arm region of the prodomain distant from the proteolytic cleavage sites. This epitope is highly sequence-divergent, having only limited similarity to other closely related members of the transforming growth factor β superfamily. Hydrogen/deuterium exchange MS experiments indicated that antibody binding induces conformational changes in pro- and latent myostatin that span the arm region, the loops contiguous to the protease cleavage sites, and the latency-associated structural elements. Moreover, negative-stain EM with full-length antibodies disclosed a stable, ring-like antigen–antibody structure in which the two Fab arms of a single antibody occupy the two arm regions of the prodomain in the pro- and latent myostatin homodimers, suggesting a 1:1 (antibody:myostatin homodimer) binding stoichiometry. These results suggest that SRK-015 binding stabilizes the latent conformation and limits the accessibility of protease cleavage sites within the prodomain. These findings shed light on approaches that specifically block the extracellular activation of growth factors by targeting their precursor forms.

## Introduction

Myostatin (growth/differentiation factor 8 (GDF8); gene name *MSTN,* gene ID 2260) is a member of the transforming growth factor β (TGF-β)^3^ superfamily of signaling proteins that is primarily expressed in skeletal muscle, with low-level expression reported in adipose and cardiac tissues (1, 2). Myostatin functions as a negative regulator of skeletal muscle growth, and its overexpression results in a substantial decrease in skeletal muscle mass (3, 4). Conversely, genetic deficiencies in myostatin in humans and other species result in significant increases in muscle mass with few apparent detrimental effects (1, 5–7). Thus, inhibition of myostatin signaling provides a therapeutic opportunity to impact diseases of muscle wasting.

**Figure 1.**
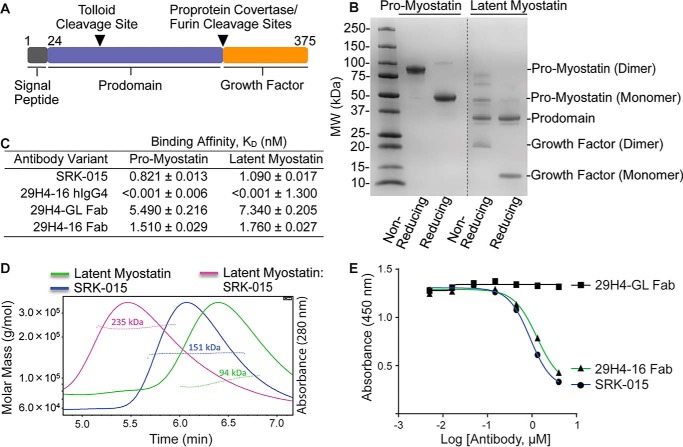
**SRK-015 and analogs bind and block proteolytic activation of myostatin.** *A*, linear cartoon of pro-myostatin showing the signal peptide (*black*), prodomain (*blue*), and growth factor (*orange*). The tolloid and furin protease cleavage sites are indicated by *inverted triangles. B*, profile of Expi293F-expressed and purified pro- and latent myostatin run under nonreducing and reducing SDS-PAGE. *MW*, molecular weight. *C*, relative binding affinities of SRK-015 and analogs to pro- and latent myostatin, determined using biolayer interferometry (FortéBio Octet). Data are presented as mean ± S.E. of duplicate measurements. *D*, SEC-MALS profiles of latent myostatin, SRK-015, and the latent myostatin–SRK-015 complex. The *dotted lines* across the peaks represent the molar mass of the samples determined from the light scattering data. *E*, ELISA-based myostatin activity assay showing that SRK-015 and analogs block proteolytic activation of myostatin. The data are representative of three independent experiments.

Like other members of TGF-β superfamily, myostatin is expressed as an inactive precursor, characterized by the presence of an N-terminal signal peptide and a prodomain that holds the growth factor in an inactive conformation (Fig. 1*A*). Upon cleavage of the N-terminal peptide, the secreted pro-myostatin containing the prodomain and growth factor remains intact. The activation mechanism of pro-myostatin involves two distinct proteolytic cleavage events. Furin-like proprotein convertase cleaves an R*XX*R sequence motif between the prodomain and growth factor, yielding a latent, noncovalent complex of the prodomain and growth factor (latent myostatin) that remains unable to bind its receptor (8–11). Subsequent proteolytic cleavage within the prodomain by a member of the BMP1/tolloid family of metalloproteases activates the latent myostatin complex (11, 12), destabilizing the prodomain–growth factor interface and releasing the growth factor (13). The mature growth factor is then poised to bind cell surface receptors, including activin-responsive type II (ActIIRA or ActIIRB), activin type I (ALK4), and TGF-β type I (ALK5), and induce downstream signaling (10, 14, 15).

Recent structural characterization of pro- and latent myostatin demonstrated a homodimeric structure with an open, V-shaped conformation (13, 16, 17) that is distinct from the ring-shaped, cross-armed structures of pro- and latent TGF-β1 (13, 18, 19). This open conformation is structurally similar to the open configurations of the related TGF-β family members proactivin A (13, 20) and pro-BMP-9 (13, 21). By comparing these structures, insights into how the prodomain confers latency to the precursor forms can be inferred. The latency can be attributed to structural elements at the binding interface that stabilize the prodomain–growth factor interaction. These regions include the α1 helix, latency lasso, and β1 strand in the prodomain and the β6′ and β7′ strands in the growth factor (13, 16, 17, 19, 22). This differs from proactivin A and pro-BMP9, where equivalent signaling activity has been shown between the precursor and mature growth factor (20, 23), suggesting that the prodomain has an insignificant inhibitory role in these systems.

Multiple inhibitors of myostatin signaling have been discovered and are in various phases of drug development for muscle disorders (24–26). The majority of these inhibitors interfere with the ability of the mature growth factor to bind to its cognate receptor by directly binding to the growth factor (27–29) or receptor (30, 31) or acting as ligand traps (32). Follistatin, a natural inhibitor of myostatin, has also been used as a therapeutic agent (33, 34). Although encouraging results have been reported from these approaches, the high sequence homology of the mature growth factor among the TGF-β superfamily (35, 36) and the redundant use of receptors for multiple growth factors (37–39) may pose potential clinical challenges brought about by lack of target specificity (26, 32, 40). Thus, an innovative approach that confers efficacy, target specificity, and the potential for enhanced safety is desired for effective therapeutic agents to treat muscle wasting.

The prodomain region has the most sequence diversity among TGF-β family members (35, 36) and is therefore a promising region to target to achieve specificity using antibodies. We have previously identified antibodies that are highly specific for pro- and latent myostatin and prevent activation of the latent form (41). SRK-015 is an optimized variant of the parental 29H4 antibody. Both antibodies bind to pro- and latent myostatin with high specificity and inhibit activation of latent myostatin by preventing tolloid-mediated proteolytic cleavage (41). Treatment with SRK-015 showed significant increases in muscle mass and function in healthy mice and in a preclinical mouse model of dexamethasone-induced muscle atrophy (41). Furthermore, a variant of SRK-015 containing mouse constant regions, muSRK-015P, effectively increased muscle mass and function and improved bone phenotype in a mouse model of spinal muscular atrophy (42). Currently, SRK-015 is in clinical development for treatment of spinal muscular atrophy.

In this study, integrated structural and biochemical approaches were used to understand the basis of the specific recognition and binding of SRK-015 to pro- and latent myostatin. These data allowed us to interrogate the inhibitory mechanism of SRK-015 in preventing proteolytic activation. The resulting cocrystal structure and in-solution dynamics studies by hydrogen/deuterium exchange MS (H/DX-MS) revealed key insights into the specificity of SRK-015 to the precursor forms of myostatin. The results identified a conformational epitope in the arm region of the prodomain. In addition, changes in conformational dynamics to regions distant from the epitope were observed. Binding of SRK-015 results in decreased solvent accessibility in regions including the latency-associated structural elements and the loops contiguous to the protease cleavage sites. Furthermore, negative-stain EM revealed the precise binding orientation of SRK-015 to both pro- and latent myostatin, demonstrating a stable ring-like conformation of the antibody–antigen complex. Taken together, these findings elucidate the molecular details of a novel strategy for specific inhibition of growth factor activation by targeting the precursor forms.

## Results

### SRK-015 and related analogs bind and prevent pro- and latent myostatin activation

A phage-based discovery campaign aimed at identification of inhibitory antibodies targeting myostatin precursors yielded a number of specific antibodies capable of preventing proteolytic activation (41). One of these antibodies, 29H4, was further optimized for affinity and germline content. SRK-015, also called 29H4-GL, is a variant of the parental 29H4 antibody in which all non-germline amino acids within the framework regions were replaced with the amino acids present in the germline gene. SRK-015 was constructed on a human IgG4 framework containing the Adair hinge mutation (S228P) (43) to reduce chain swapping, which can be observed for native IgG4 antibodies (44). 29H4-16 is a high-affinity variant of the parental 29H4 antibody and contains four amino acids changes within the complementarity-determining regions (CDRs) of both the light and heavy chains.

To understand the structural basis of the binding mechanism of SRK-015 to pro- and latent myostatin, biochemical and structural approaches were employed. Human pro- and latent myostatin constructs were generated and expressed in mammalian Expi293F^TM^ cells. The secreted proteins were purified using immobilized nickel (II) affinity and gel filtration chromatography. For crystallographic constructs, mutations in the tolloid (D99A) and furin (R263A and R266A) protease cleavage sites were introduced to generate an uncleavable precursor protein, pro-myostatin. Pro-myostatin runs as single bands at approximate sizes of 83 and 41 kDa under nonreducing and reducing SDS-PAGE conditions, respectively (Fig. 1*B*). The 83-kDa band represents the homodimeric, disulfide-linked form of pro-myostatin that dissociates to a 41-kDa monomeric form under reducing conditions. The latent myostatin is devoid of mutations in the tolloid/furin protease cleavage sites; thus, under nonreducing and reducing conditions, several cleavage products were visible, consistent with the presence of unprocessed precursor forms of myostatin, the prodomain, and the growth factor, which are noncovalently associated.

Using the recombinantly expressed and purified proteins, the binding affinities of 29H4 and derivative antibodies to pro- and latent myostatin were tested using biolayer interferometry (Fig. 1*C*). The antibody variants were tested as full-length IgG4 and as Fab fragments. Full-length SRK-015 showed an approximately nanomolar binding affinity to both pro- and latent myostatin and an approximate 7-fold decrease in binding affinity in its Fab version. 29H4-16 hIgG4 demonstrated high-affinity binding to both pro- and latent myostatin. In fact, the dissociation constant (*K_D_*) is not reliably quantifiable, as the off rate of the antibody was observed to be below the limit of sensitivity of the biolayer interferometry Octet instrument. Monomeric 29H4-16 Fab showed a decrease in binding affinity compared with its IgG4 form, a similar trend as observed for SRK-015.

Further, the stoichiometry of binding of SRK-015 to latent myostatin was investigated. Size-exclusion chromatography (SEC) coupled to multi-angle light scattering (MALS) was used to determine the absolute molar mass of the latent myostatin, SRK-015, and the latent myostatin–SRK-015 complex. The latent myostatin and SRK-015 were each eluted as a single peak with homogenous mass distribution (Fig. 1*D*). The observed average molar masses for latent myostatin and SRK-015 were 94 and 151 kDa, respectively, which are close to their calculated molar masses (82 and 145 kDa, respectively). Moreover, the latent myostatin–SRK-015 complex showed a homogenous mass distribution with an average molar mass of 235 kDa, suggesting that one molecule of SRK-015 binds one latent myostatin homodimer, consistent with a 1:1 binding stoichiometry.

Similar to other members of the TGF-β superfamily of growth factors, pro-myostatin is activated by extracellular proteases, including proprotein convertase (Furin or PCSK5) (5, 10, 45, 46) and tolloid metalloproteases (mTTL2 or BMP-1) (11, 12, 45–48). Cleavage by these proteases releases the growth factor from the prodomain cage, where it eventually binds to the cell surface receptors, *e.g.* type 1 (ALK4/5) and type II (ActRIIa/b) receptors (1). To determine the effect of the antibodies on proteolytic processing of latent myostatin, a receptor engagement assay was developed using an extracellular domain of human ActRIIb fused to the Fc region of the mouse IgG1 (ActRIIb-Fc) as a reagent to capture the released growth factor. In this assay, SRK-015 or antibody fragments of 29H4-GL and 29H4-16 were first incubated with latent myostatin and further incubated with conditioned medium containing mTLL2 tolloid metalloprotease. Released myostatin growth factor bound to the receptor was detected using an antibody directed to the mature growth factor, which had an epitope so that it could be measured in the presence of the receptor fusion. The results from this receptor engagement assay showed a dose-dependent decrease in the amount of released myostatin growth factor bound to the ActRIIb-Fc when incubated with SRK-015 or 29H4-16 Fab (Fig. 1*E*), suggesting that both antibodies effectively block mTLL2 tolloid–mediated proteolytic activation and that 29H4-16 is a suitable surrogate for biochemical and structural studies. Monomeric 29H4-GL Fab had no effect on blocking mTLL2-mediated proteolytic activation of latent myostatin, which can be attributed to its weaker binding affinity to latent myostatin (Fig. 1*C*) and suggests that avidity may play a role in the potent inhibition observed for the bivalent antibody.

### The cocrystal structure of pro-myostatin:29H4-16 Fab reveals the specificity of binding to the arm region in the prodomain

To gain further structural insights into the binding mechanism of SRK-015 to pro- and latent myostatin, the X-ray cocrystal structure of pro-myostatin–29H4-16 Fab was obtained. The structure was solved by molecular replacement using search models derived from crystal structures of Fab (PDB codes 5GGU and 5F3H), myostatin (PDB code 3HH2), and pro-TGFβ1 (PDB code 3RJR). Subsequently, publication of the pro-myostatin structure (PDB code 5NTU) allowed further improvement in many regions of the structure. The structure was completed after successive rounds of manual building and refinement using Refmac5 (49) to a final resolution of 2.79 Å with *R*_work_ and *R*_free_ values of 22% and 26%, respectively, and belongs to space group P2_1_2_1_2_1_ (Table S1).

**Figure 2.**
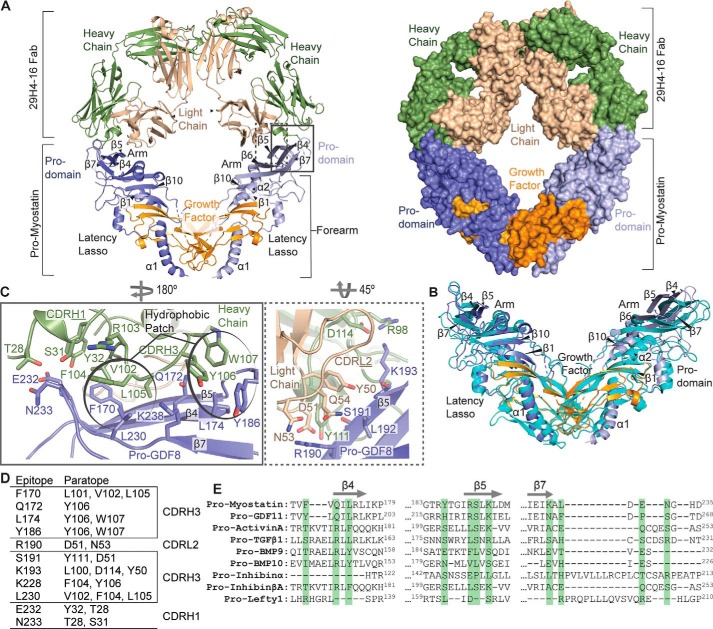
**Cocrystal structure of pro-myostatin–29H4-16 Fab.** *A*, the overall structure of the pro- myostatin–29H4-16 Fab complex, showing two 29H4-16 Fabs occupying the arm regions in the prodomain of the pro-myostatin homodimer. The structure of the complex is shown in ribbon (*left panel*) and surface (*right panel*) representations. *B*, structural alignment of the 29H4-16 Fab–bound pro-myostatin structure (similar color scheme as in *A*) with the previously reported crystal structure of unbound pro-myostatin (*cyan*) (PDB code 5NTU). *C*, the binding interface of the pro-myostatin–29H4-16 complex, highlighting the key amino acid residues involved in the interaction. The *left* and *right panels* represent close-up views of the amino acid residues in the CDRs of the heavy (*green*) and light (*light yellow*) chains, respectively, that are in contact with the amino acid residues in the arm region of the pro-myostatin homodimer. The amino acid residues that cluster as hydrophobic patches in the binding interface are *circled. D*, summary of amino acid residues in the epitope and paratope at the binding interface of the pro-myostatin–29H4-16 Fab complex. *E*, sequence alignment of the regions (β4, β5, and β7) that covered the conformational epitope in pro-myostatin across related members of the TGF-β superfamily. Highlighted in *green* are the amino acid residues comprising the epitope in pro-myostatin.

In the observed crystal form, pro-myostatin exists as a homodimer that sits on the crystallographic two-fold axis so that the asymmetric unit contains one pro-myostatin monomer and one Fab, whereas the entire complex maintained a 2:2 (pro-myostatin monomer:Fab) binding stoichiometry (Fig. 2*A*). Overall, 29H4-16 Fab binds to the arm region in the prodomain, with one Fab occupying each arm region of the pro-myostatin homodimer. The 29H4-16 Fab-binding region in the arm is relatively distant from the loops containing the furin and tolloid protease cleavage sites. This was unexpected, given that the antibody inhibits tolloid protease–mediated activation of the myostatin precursor (Fig. 1*E*).

The pro-myostatin structure reported in this study contains several regions with unresolved electron densities, similar to the previously published structure of unbound pro-myostatin (16). The regions with missing density include the N-terminal prodomain sequence (Asn^24^-Ala^41^), the loops containing the tolloid (Val^96^-Asp^108^) and furin (Thr^260^-Gly^269^) cleavage sites, a region in the prodomain (Ser^125^-Pro^135^), and a region in the growth factor (Gly^311^-Pro^338^). As the electron density for the loops that contain the protease cleavage sites are among the missing regions in both structures, the relative conformations of these loops between the unbound and antibody-bound states of pro-myostatin are unresolved.

The binding of 29H4-16 in the arm region of the prodomain suggests two potential mechanisms of inhibition that cannot be distinguished by the crystal structure alone: steric blockade of the protease preventing access to the proteolytic cleavage site or antibody binding eliciting conformational changes in the loop adjoining the proteolytic cleavage site, making this loop less accessible to proteolytic processing. Structurally, the 29H4-16 Fab showed an expected canonical immunoglobulin fold made of four β-sandwich domains. Pro-myostatin shows the typical V-shaped conformation (16) (Fig. 2*A*) seen in other TGF-β superfamily members but is structurally different from the ring-shaped, cross-armed structure of pro-TGF-β1 (22). Pro-myostatin shares similar structural elements with the other members of TGF-β family, including an *N*-linked prodomain, which, in coordination with its latency lasso and the arm region, holds the C-terminal growth factor domain in place.

Structural alignment of the pro-myostatin–29H4-16 Fab complex with the previously published unbound pro-myostatin (PDB code 5NTU) (16) (Fig. 2*B*) showed a root mean square deviation of 3.03 Å, indicating that the Fab binding elicits conformational changes in the pro-myostatin structure. Particularly the loops and β strands in the arm region of pro-myostatin shift to accommodate the loops of the CDRs of 29H4-16 Fab. Additionally, as part of the global adaptation to 29H4-16 binding, structural elements within the prodomain (α1 helix, latency lasso, α2 helix, and β1 strand) and part of the growth factor displayed changes in conformation between the unbound and 29H4-16 Fab–bound states of pro-myostatin.

The binding interface of pro-myostatin and 29H4-16 involves two CDRs from the heavy chain (CDRH1 and CDRH3) and one CDR from the light chain (CDRL2) of the 29H4-16 Fab and the arm region within the prodomain of pro-myostatin spanning the β4, β5, and β7 strands (Fig. 2*C*). The pro-myostatin–29H4-16 Fab binding interface buries 4.2% (1650 Å^2^) of the total solvent-accessible surface area (19,897 Å^2^ per pro-myostatin monomer and 19,645 Å^2^ for the Fab), which is within the typical range for antibody–antigen interactions (50). Moreover, the heavy and light chains of 29H4-16 Fab span a total buried surface area per monomer of pro-myostatin of 1306 and 429 Å^2^, respectively. This suggests that 75% of the contact surface area of 29H4-Fab to pro-myostatin is contributed by the heavy chain. The interface is made up of hydrophobic, hydrogen-bonding, and salt bridge interactions. Hydrophobic patches, including aromatic stacking interactions, contribute 45% of the contact residues in the binding interface. The hydrophobic patch is composed of pro-myostatin residues Phe^170^, Leu^174^, Tyr^186^, and Leu^230^ and CDRH3 residues Leu^101^, Val^102^, Phe^104^, Leu^105^, Tyr^106^, and Trp^107^. Amino acid residues of CDRH3 (Leu^100^, Phe^104^, Tyr^106^, and Asp^114^), CDRH1 (Thr^28^, Ser^31^, and Tyr^32^), and CDRL2 (Asp^51^ and Asn^53^) interact via H-bonding with the residues (Gln^172^, Arg^190^, Ser^191^, Lys^193^, Lys^228^, Glu^232^, and Asn^233^) in the arm region of pro-myostatin. Additionally, Asp^114^ from CDRH3 and Asp^51^ from CDRL2 form salt bridges with residues Arg^190^ and Lys^193^ in the β5 strand of pro-myostatin. The nonlinear distribution of the contact residues in pro-myostatin shows that 29H4-16 binds a conformational epitope. The amino acid residues comprising the epitope–paratope of the pro-myostatin–29H4-16 binding interaction are summarized in Fig. 2*D*.

Sequence alignment of the conformational epitope of 29H4 in pro-myostatin across relevant members of TGF-β superfamily showed significant sequence diversity in this region (Fig. 2*E*). The low sequence conservation comprising the epitope supports previously reported data showing the specificity of SRK-015 over the other relevant members in the TGF-β family, including pro-GDF11, proactivin A, BMP-9, BMP-10, and pro-TGFβ1 (41).

### SRK-015 and related antibody binding evoked changes in conformational dynamics of pro- and latent myostatin spanning the arm, protease cleavage sites, and latency-associated structural elements

The activation pathway of pro-myostatin involves cleavage by furin and tolloid proteases, which releases the growth factor for downstream signaling. Importantly, a pool of extracellular, uncleaved pro-myostatin has been observed in skeletal muscle in addition to furin-cleaved, latent myostatin, which predominates in serum (3, 41, 45, 47). Thus, in the context of accessibility to extracellular modulators, *e.g.* neutralizing antibodies, both precursor forms (pro- and latent) are relevant. To gain additional structural insights into the binding of SRK-015 and the variant 29H4-16 antibody to the pro-myostatin and latent myostatin forms, the in-solution conformational changes accompanied by these binding interactions were measured using H/DX-MS. This method relies on exchange of the protein backbone amide hydrogens with deuterium in solution, in which the rate of H/D exchange depends on solvent accessibility for a given region. Thus, the structural and regional dynamics of the protein play a large role in the rate of exchange (51). For example, regions at the core of a protein structure or occluded by ligand binding exhibit lower measurable H/D exchange than solvent-accessible regions, and information regarding protein conformational dynamics and protein–protein interactions (*e.g.* antigen–antibody) can be generated.

H/DX-MS experiments were performed on pro- and latent myostatin in its unbound and 29H4-(GL/16) Fab-bound states. The protein samples were incubated in deuterated buffer between 10 s and 1 h for H/D exchange, followed by on-column pepsin digestion and MS for peptide mass analysis. The peptic peptide coverage was found to be 94.2% and 88.0% across the linear amino acid sequence for pro-myostatin and latent myostatin, respectively (Fig. S1). The regions with no peptide coverage are assigned to regions within the growth factor that contain disulfide-bonded cysteines, suggesting a very stable region recalcitrant to reduction and protease digestion. Heatmaps representing the relative differential deuteration profiles of pro- and latent myostatin in the unbound and Fab-bound states over the course of H/D exchange are illustrated (Fig. S2) to provide the overall conformational flexibility profile across regions of the protein. As expected, in both pro- and latent myostatin, the buried regions in the core of the protein structure showed lower H/D exchange levels than the solvent-exposed or flexible regions of the protein. The peptic peptides with higher H/D exchange are localized within regions in the prodomain, including the N-terminal association region, latency lasso, α2 helix, and the top of the arm region comprising the loop region adjoining the β5 strand. Relatively higher H/D exchange events have also been observed in loop regions contiguous to the furin and tolloid protease cleavage sites. These data suggest that the higher conformational flexibility of these regions could be essential for the structural and dynamic regulation of pro- and latent myostatin as well as for its accessibility to proteolytic cleavage for activation.

**Figure 3.**
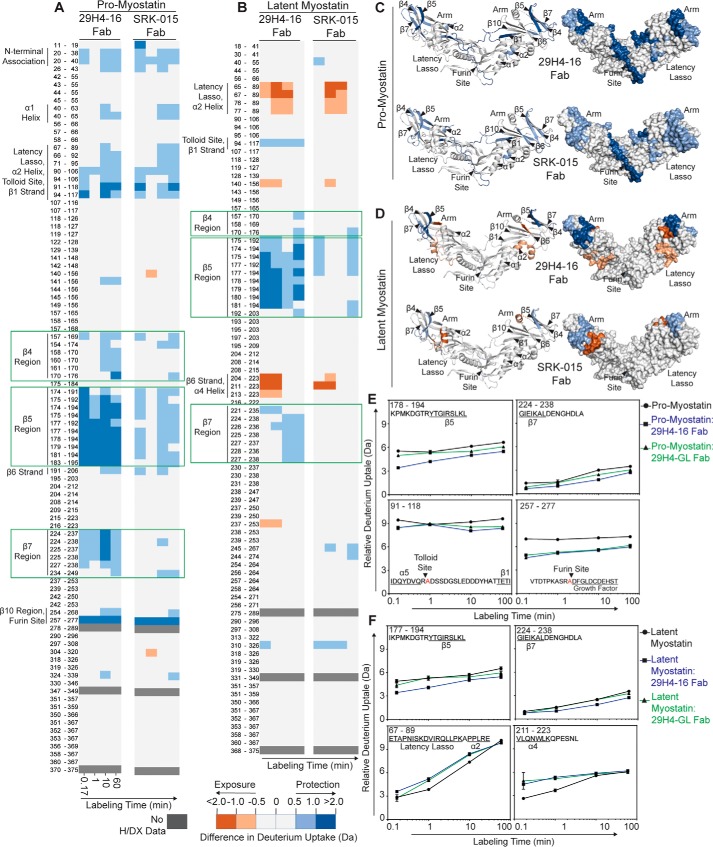
**Fluctuations in conformational dynamics of pro- and latent myostatin upon binding to antibody fragments of SRK-015 and 29H4-16 probed by H/DX-MS.** *A* and *B*, heatmap showing the differences in deuterium uptake of peptic peptides identified in pro-myostatin (*A*) and latent myostatin (*B*) between its unbound and Fab-bound (SRK-015 Fab or 29H4-16 Fab) states at the indicated time of exposure to deuterated solvent. The amino acid residues for each peptic peptide are listed, and the identities of the structural elements that showed significant difference in H/D exchange between the unbound and Fab-bound states of pro- and latent myostatin are indicated. The peptic peptides covering the epitope regions are highlighted by *green boxes. C* and *D*, the significant difference in H/D exchange profiles between the unbound and Fab-bound states of pro-myostatin (*C*) and latent myostatin (*D*) after 10 min of labeling are mapped onto the model structure of pro-myostatin (PDB code 5NTU) in ribbon (*left panel*) and surface (*right panel*) representations. For these data, a deuterium uptake difference of more than 0.5 Da is considered significant at a 98% confidence interval, calculated as described previously (66). The intensities of the *blue* and *red colors* represent peptides that undergo either a significant decrease (less solvent exposure/less flexible) or increase (more solvent exposure/more flexible), respectively, during H/D exchange events between the unbound and the Fab-bound states of pro- and latent myostatin. *E* and *F*, representative deuterium incorporation plots of key peptic peptides that showed significant H/D exchange between the unbound and Fab-bound states of pro-myostatin (*E*) and latent myostatin (*F*). These peptide regions covered the epitope in the arm of the prodomain, the protease cleavage sites, and the structural elements associated with myostatin latency. Figs. S3 and S4 include all deuterium incorporation plots from these studies. *Error bars*, S.D. of duplicate H/DX-MS measurements done on two separate days.

Significant H/D protection was observed for several peptic peptides in pro- and latent myostatin upon binding to 29H4-(16/GL) Fabs. These regions included the top of the arm of the prodomain, including the amino acid residues adjoining the β4 (170–176), β5 (174–195), and β7 (234–238) strands (Fig. 3). The representative deuterium uptake plots for the peptide regions in the arm of the prodomain that showed significant protection from H/D exchange are shown for pro-myostatin (Fig. 3*E*) and latent myostatin (Fig. 3*F*). The deuterium uptake plots of all resolved peptic peptides for pro-myostatin and latent myostatin are shown in Figs. S3 and S4. Overall, the binding of 29H4-16 Fab resulted in a higher extent of significant H/D protection across regions of pro- and latent myostatin than 29H4-GL Fab (Fig. 3), which can be attributed to the higher affinity of the 29H4-16 Fab variant to both forms of myostatin.

The H/DX-MS dynamic studies of the Fab bound complexes revealed multiple protected regions in the arm of the prodomain, which is in agreement with the result of the conformational epitope identified in the cocrystal structure of pro-myostatin–29H4-16 Fab (Fig. 2). Additionally, significant changes in the H/D exchange profiles were also observed in regions distant from the epitope in the arm region of the prodomain covering the tolloid (peptide 91–118) and furin (peptide 257–277) cleavage sites in pro-myostatin that showed an overall decrease in H/D exchange upon binding to 29H4-(16/GL) Fabs (Fig. 3, *A*, *C*, and *E*). The observed decrease suggests that the peptic peptide regions containing the proteolytic cleavage sites become less flexible and less solvent-exposed when bound to 29H4-(16/GL) Fabs. In contrast, the corresponding peptides containing the tolloid/furin protease cleavage sites in latent myostatin displayed no significant changes in H/D exchange profile upon binding to 29H4-(16/GL) Fabs (Fig. 3, *B*, *D*, and *F*). This suggests that the overall flexibility of these regions is not affected in the presence of the antibody fragments. The differences in H/D exchange pattern in the region contiguous to the tolloid and furin cleavage sites between the pro- and latent myostatin indicate that these two forms are in distinct conformational states before and after furin cleavage.

Pro- and latent myostatin showed additional differences in H/D exchange profiles in key structural elements upon binding to 29H4-(16/GL) Fab. In pro-myostatin, a significant decrease in H/D exchange was observed in the regions of the N-terminal association site, α1 helix, latency lasso, α2 helix, and β1 and β10 strands (Fig. 3*A*), suggesting that these regions become less flexible in both complexes. On the other hand, latent myostatin showed increased H/D exchange in the latency lasso region, part of the β6 strand, and α4 helix, indicative of enhanced solvent exposure and conformational flexibility of these regions when bound to 29H4-(16/GL) Fabs (Fig. 3*B*). The antibody-mediated changes in overall conformational flexibility of regions adjoining the α1 helix, latency lasso, α2 helix, and β1 strand in pro- and latent myostatin are particularly interesting because these regions are proposed to be key in maintaining myostatin latency (13, 16).

Altogether, the dynamics of pro- and latent myostatin suggest that binding of SRK-015 and the antibody variant 29H4-16 induced a decrease in the conformational flexibility in the antibody epitope in the arm region of the prodomain. Binding of the antibody also evoked a decrease in solvent exposure of structural elements that are important for maintaining pro-myostatin latency, stabilizing the prodomain–growth factor interaction. Likewise, binding of the antibody resulted in a decrease in solvent exposure of the tolloid and furin protease cleavage sites, which may suggest limited access of these sites to proteases for conversion of pro-myostatin to latent myostatin.

### Negative-stain EM revealed the binding orientation and stoichiometry of SRK-015 to pro- and latent myostatin

**Figure 4.**
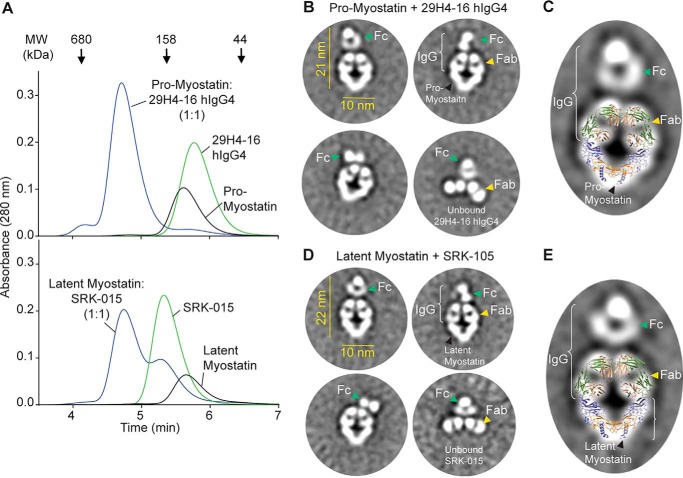
**Negative-stain EM revealed the structural features of pro- and latent myostatin in complex with 29H4-16 hIgG4 and SRK-015, respectively.** *A*, SEC profile to confirm the complex formation of pro-myostatin–29H4-16 hIgG4 and latent myostatin–SRK-015 prior to negative-stain EM. *B* and *D*, representative EM class averages for pro-myostatin–29H4-16 hIgG4 (*B*) and latent myostatin–SRK-015 (*D*). Also observed in the class averages are unbound full-length antibodies. *C* and *E*, the coordinates from the X-ray cocrystal structure of pro-myostatin–29H4-16 Fab (Fig. 2*A*) showed a good fit for the density map of a representative class average of pro-myostatin:29H4-16 hIgG4 (*C*) and latent myostatin–SRK-15 (*E*). A broader set of class averages is included in Fig. S5.

To visualize the overall molecular organization and precise orientation of SRK-015 binding to pro- and latent myostatin, negative-stain EM was conducted using pro-myostatin and latent myostatin incubated with full-length monoclonal antibodies, 29H4-16 hIgG4, and SRK-015, respectively. Prior to conducting negative-stain EM, the samples were subjected to size-exclusion chromatography to ensure that complex formation of full IgGs to pro- and latent myostatin could be observed (Fig. 4*A*). The antigen–antibody complexes were then stained with uranyl formate for negative stain EM imaging. Further 2D class averages obtained for pro-myostatin–29H4-16 IgG4 (Fig. 4*B* and Fig. S5*A*) and latent myostatin–SRK-015 (Fig. 4*D* and Fig. S5*B*) showed limited but well-defined orientations of the particles representing the antigen–antibody complex. In fact, only two rounds of 2D class averaging were necessary for both antigen–antibody samples because of the observed sample homogeneity.

Representative 2D class averages for pro-myostatin–29H4-16 hIgG4 (Fig. 4*B*) and latent myostatin–SRK-015 (Fig. 4*D*) showed particles that represent the antigen–antibody complex as well as the unbound full-length mAb. The samples were combined at a 1:2 (antigen:antibody) molar ratio to achieve a saturating condition; hence, there were no observed class averages that could represent the unbound pro- or latent myostatin homodimers. The class averages representing the antigen–antibody complexes showed lengths of about 21–22 nm with an average width of 10 nm. The electron density displaying the relative orientations of the pro- and latent myostatin dimers and the Fab and Fc arms of the antibody could be readily identified. Visible in the class averages are the two Fab arms of the antibody occupying the two arm regions of the prodomain for the pro- and latent myostatin homodimers, suggesting a 1:1 binding stoichiometry of the antigen–antibody complex. The pro- and latent myostatin homodimers adopted a V-shaped conformation with an angle of ∼80° between the two arms of the prodomain. This angle is conserved across class averages and between the two different antigen–antibody samples, suggesting that there is minimal flexibility around the dimer interface of both myostatin samples when bound to the antibody. In contrast, the Fc region showed a less well-defined density with variable orientations relative to the rest of the structural organization of the complex, suggesting a conformationally elastic domain that is likely a consequence of flexibility around the hinge region of the antibody.

The superposition of the cocrystal structure of pro-myostatin–29H4-16 Fab (Fig. 2*A*) into the representative 2D class averages for pro-myostatin–29H4-16 hIgG4 (Fig. 4*C*) and latent myostatin–SRK-015 (Fig. 4*E*) showed a strikingly similar overall structural organization, with the exception of the presence of an additional density assigned to the Fc region of the full length antibody. Taken together, these data suggest the utility of an integrated structural approach to map out the details of the binding mechanism and stoichiometry as well as the mechanism of action of SRK-015, a neutralizing antibody against pro- and latent myostatin.

## Discussion

The structural characterization data described here reveal the molecular mechanism of a novel strategy to inhibit myostatin signaling by preventing proteolytic activation of the myostatin precursors. This strategy is distinct from other approaches that target the interaction of the mature growth factor with its receptor (27, 29, 31, 32, 52–55). SRK-015 and its high-affinity analog 29H4-16 showed highly specific binding to the arm region in the prodomain of pro/latent myostatin and influenced the conformational flexibility of known latency-associated structural elements as well as regions contiguous to the tolloid and furin proteolytic cleavage sites. These data suggest the potential mechanism of action of SRK-015 and its analog, which, upon binding to the arm region, promotes stabilization of the prodomain–growth factor interaction and preserves the state of latency of the myostatin precursors. In addition, the allosteric conformational changes in the loops adjoining the tolloid and furin sites suggest that these proteolytic cleavage sites are upheld in a conformation less accessible to protease for activation.

To date, the pro-myostatin–29H4-16 Fab cocrystal structure represents the first crystal structure of an antibody bound to the proform of myostatin. More importantly, SRK-015 and related analogs represent first-in-class antibodies that recognize both prodomains in the symmetrical V-shaped structure of the precursor myostatin homodimer. This precise binding orientation was confirmed by negative-stain EM showing that both Fab arms of a single antibody occupied the prodomains of one pro- or latent myostatin dimer forming a stable ring-like antigen–antibody complex. This unique anchoring mechanism of SRK-015 could be attributed to the distinct set of complementary residues in the binding interface as well as the avidity observed because of the bivalency of the IgG4 framework.

Observed in the cocrystal structure and by H/DX-MS, binding of SRK-015 and the high-affinity 29H4-16 analog elicits changes in conformation and solvent accessibility to regions distant from the epitope, including the latency-associated structural elements that are crucial for the overall activation mechanism of myostatin. One distinguishing feature in the prodomains of TGF-β superfamily members is the N-terminal helix–loop–helix motif in the forearm region comprised of residues 42–115, which covers regions in the α1 helix, latency lasso, and α2 helix. This peptide region was previously identified as an inhibitory fragment of pro-myostatin (8). In the crystal structure of pro-myostatin, these latency-associated regions assemble into a straightjacket-like structure that wraps around the growth factor and stabilizes the prodomain–growth factor interface. Interestingly, the region of the α2 helix is uniquely positioned, facing the growth factor and occluding the putative type II receptor (ActRIIa/b) sites. Several others have made inhibitory peptides and mutations in the conserved aliphatic residues within the α1 helix, a region that is buried in the hydrophobic groove of the growth factor, and have shown that this region contributes to latency and affects the function of myostatin (17, 56, 57). Remarkably, in previous H/DX-MS studies, tolloid cleavage of latent myostatin has resulted in higher conformational flexibility in the latency-associated structural elements within the prodomain (α1 helix, latency lasso, and β1 strand) and growth factor (β6′ and β7′ strands) that primes the latent myostatin complex for dissociation (13). This priming event leads to destabilization of the prodomain–growth factor interface. Together, the observed changes in the conformations and solvent accessibility of the latency-associated structural elements suggest that binding of SRK-015 stabilizes the conformation of both myostatin precursors, including the pro-myostatin form, which represents the dominant extracellular form in skeletal muscle (41, 45), preventing further activation.

The structure of the loops adjacent to the tolloid and furin cleavage sites inform the accessibility of these sites for proteolytic processing, a critical step toward myostatin activation. Although the structure of the loop contiguous to the furin cleavage site is known (16), the structures of pro-myostatin reported in this paper and by others (16) have shown consistent lack of reliable electron density around the tolloid cleavage site, indicative of a highly disordered region. Thus, the H/DX-MS data showing the relative conformational flexibility around the tolloid and furin cleavage sites provide structural insight into these regions. Binding of the antibody fragments of SRK-015 and its high-affinity analog, 29H4-16, evoked a decrease in H/D exchange events in the regions covering the tolloid and furin sites of pro-myostatin, suggesting that these regions become less solvent-exposed and less flexible. The restricted conformational flexibility around these protease sites may result in limited access of these sites to proteases, preventing further proteolytic activation of pro-myostatin. The structural data presented here are consistent with the *in vitro* proteolytic inhibition assay shown in this work (Fig. 1*E*) and further validated by previous *in vitro* and *in vivo* assays showing that SRK-015 and analogs prevent tolloid-mediated cleavage of myostatin precursors (41).

Additionally, the H/DX-MS dynamics studies of myostatin precursors were able to resolve representative peptides covering the N-terminal association region, which is typically not resolved in the crystal structures of pro-myostatin. This region in the prodomain is an important structural feature of the TGF-β family, as it serves as an anchor point for various presenting molecules for storage in the extracellular matrix or for cell surface expression (36). The myostatin precursors have been suggested to noncovalently associate with presenting molecules in the extracellular matrix, such as latent TGF-β–binding protein 3 (LTBP3) (45) and perlecan (58), possibly via the N-terminal association region. However, the biological relevance of such interactions remains unclear. Results from the current H/DX-MS study showed that SRK-015 binding evoked a significant decrease in H/D exchange in the N-terminal association region specific to the pro-myostatin form (Fig. 3*A*), suggesting that this region becomes less flexible upon antibody binding, which could impact deposition of myostatin into the extracellular matrix.

The discovery of SRK-015 and related antibodies opens opportunities for specific therapeutic intervention for myostatin signaling. Previous clinical approaches that inhibit myostatin signaling were directed to block the growth factor–receptor interaction and included binders of the growth factor (59) or receptor (40), a ligand trap (32), and use of the natural inhibitor of the myostatin growth factor, Follistatin (33, 34). However, because of the high sequence homology of mature growth factors (35, 36) and the propensity of the receptors to accommodate multiple growth factors (37–39), several of these myostatin signaling–directed therapeutic agents showed cross-reactivity toward other members of the TGF-β superfamily. For instance, several clinical-stage antibodies targeting the mature myostatin growth factor have been reported to cross-react with mature growth factors of activin A, BMP-9, BMP-10, and GDF11 (41). Hence, achieving a truly selective therapeutic agent via targeting the mature growth factor remains difficult. More importantly, the lack of selectivity for any therapeutic approach makes interpreting efficacy and safety data particularly challenging. For example, the ActRIIb ligand trap ACE-031 had entered clinical trials but was terminated prematurely because of toxicities such as epistaxis and telangiectasia (32). These toxicities have been postulated to be the result of overlapping specificity to BMP-9 and BMP-10 (32, 60). Thus, the unique mechanism of SRK-015 (binding specifically to the precursor forms of myostatin via the less conserved prodomain) could potentially deliver improved safety and efficacy profiles for myostatin signaling–associated therapies.

The approach of targeting the precursor forms to modulate growth factor activation could be useful in discovery of specific modulators for other members of the TGF-β superfamily or, more broadly, other signaling proteins requiring precursor processing for activation. Practically, this approach takes advantage of the sequence diversity in the prodomain relative to the growth factor domain, reducing the challenge of identifying antibodies selective for one growth factor over others that share a high level of sequence and structural identity.

Perhaps more importantly, targeting (inactive) growth factor precursors may also offer a biological advantage. Activation and signaling of the TGF-β superfamily of growth factors are local events. Growth factor activation occurs proximally to the cellular location of the growth factor receptors. In the case of myostatin, pro- and latent myostatin reside predominantly in the extracellular space within muscle fibers, in close proximity to its cognate receptors on the cell surface (41). Similarly, latent TGF-β1 can be expressed and deposited in extracellular matrix or on the surface of immune cells, depending on the cell type that produces the growth factor (61). Thus, cellular expressed TGF-β1 is often expressed on the same cell surface as the TGF-β receptors, minimizing the distance required for autocrine signaling. Therapeutic antibody blockade of the mature growth factor would require that the antibody be present at the site and time of activation to be capable of intercepting the growth factor *en route* to its receptor. Such a mechanism requires a high circulating concentration of inhibitory antibodies and an antibody with a very fast on-rate to achieve sustained functional inhibition. Preventing activation altogether substantially alleviates such requirements, reducing the bar for achieving efficacy.

In summary, use of an integrated structural approach facilitated elucidation of the molecular mechanism of a novel therapeutic strategy of targeting the precursor forms of myostatin to prevent activation and signaling for treatment of muscle-related disorders. Extracellular proteolytic processing is a common theme in the activation mechanism of members in the TGF-β superfamily. Thus, the approach of specifically targeting the precursor protein to block activation can be broadly applied to other members of the TGF-β superfamily, which may facilitate discovery of its specific modulators.

## Experimental procedures

### Construct design

The constructs for pro- and latent myostatin were cloned into the pTT5 vector using EcoRI and NotI restriction sites via Gibson assembly (New England Biolabs, E2621). Pro-myostatin was designed with an N-terminal signal peptide derived from the mouse IgG heavy chain, followed by a His_6_ tag, tobacco etch virus protease cleavage site, and myostatin amino acid residues 24–375 (Uniprot ID 014793) with furin (D99A) and tolloid (R263A and R266A) sites mutated to render it uncleavable and inactive. Latent myostatin was generated similarly with the same signal sequence, His_6_ tag, and the WT myostatin sequence (residues 24–375).

The constructs for both 29H4-GL and the 29H4-16 human IgG1 Fabs were cloned via Gibson assembly by inserting the DNA encoding the mouse Ig heavy chain V region 102 signal peptide followed by the 29H4 V_H_ and C_H_1 regions into the EcoRI and BamHI restriction sites of pTT5. The full-length IgGs of each were cloned similarly into pTT5, using identical variable domains for each of the two variants with a human IgG4 constant region with a stabilized hinge to reduce heavy-chain swapping. The 29H4-GL and 29H4-16 human λ chains were cloned via Gibson assembly by inserting DNA encoding the mouse IgG heavy chain V region 102 signal peptide followed by the 29H4-GL and 29H4-16 V_L_ and C_L_ regions into the EcoRI and BamHI restriction sites of pTT5.

### Protein expression

All antigens, full-length antibodies, and Fabs in this study were transiently expressed in Expi293F^TM^ cells (Invitrogen) using Expi293 growth medium (Invitrogen, catalog no. A1435101). For pro-myostatin used in crystallographic experiments, 2 days after initial seed culture of 0.6 × 10^6^ cells/ml, kifunensine (Tocris, catalog no. 3207) was added to the cell culture medium at a final concentration of 1 mg/liter. The transfection method described below was followed for all antigens, full-length antibodies, and Fabs. Briefly, transfection was done using polyethylenimine hydrochloride (PEI Max, Polysciences Inc., catalog no. 24765; final concentration, 2.9 mg/liter) and a total DNA amount of 1 mg/liter of culture with viable cell densities of 2–3 × 10^6^ cells/ml. The DNA:PEI solution was then added to the cell culture medium. Twenty-four hours post-transfection, the cells were supplemented with 0.5% (w/v) tryptone N1 (TekniScience Inc., catalog no. 19553) and allowed to grow for 4–5 days under optimal culture conditions (37 °C, 5% CO_2_). The cells were harvested by centrifugation at 6.9 × *g* for 20 min at 4 °C. The supernatant containing the secreted proteins was sterile-filtered using a 0.22-μm filter.

### Protein purification

Pro- and latent myostatin were subsequently purified using immobilized nickel (II) ion affinity chromatography and SEC. Briefly, the salt and pH levels of the supernatant were adjusted to 0.45 m NaCl and pH 8.0, respectively. The pH and salt-adjusted conditioned medium was loaded onto a 5-ml HiTrap Excel column (two in series, GE Healthcare) pre-equilibrated, and washed with 20 mm phosphate and 0.5 m NaCl (pH 8.0). Protein was eluted using 20 mm phosphate, 0.5 m NaCl, and 0.25 m imidazole (pH 7.8). The protein was further purified by SEC using a HiLoad 26/600 Superdex® 200 pg column (GE Healthcare) with 20 mm HEPES and 150 mm NaCl (pH 7.5) as eluent buffer. The protein was concentrated, flash-frozen in liquid nitrogen, and stored at −80 °C until use.

The antibody fragments, 29H4-16 Fab and 29H4-GL Fab, were purified by affinity binding to a 5-ml HiTrap λ Fab Select Column (GE Healthcare) in tandem with a 5-ml HiTrap Protein L Column (GE Healthcare) equilibrated and washed with 1× PBS (pH 7.4). The protein was eluted using 0.1 m H_3_PO_4_ (pH 2.7) and neutralized with 10% (v/v) 1.6 m HEPES (pH 8.0). Protein fractions were pooled and adjusted to 150 mm NaCl and sterile-filtered using a 0.22-μm filter. Pooled protein was dialyzed against 20 mm HEPES and 150 mm NaCl (pH 7.5) at 4 °C, concentrated, flash-frozen in liquid nitrogen, and stored at −80 °C until use.

The full-length antibodies, 29H4-16 human IgG4 and 29H4-GL human IgG4 (SRK-015), and ActRIIb-mouse Fc fusion protein were purified using a 5-ml HiTrap rProtein A FF Column (two in series, GE Healthcare) pre-equilibrated and washed with 1× PBS (pH 7.4). The protein was eluted using 100 mm phosphoric acid (pH 3.0) and neutralized with 10% (v/v) using 1.6 m HEPES (pH 8.0). The protein was dialyzed against 20 mm citrate and 150 mm NaCl (pH 5.5) at 4 °C, concentrated, flash-frozen in liquid nitrogen, and stored at −80 °C until use.

### Complex formation

For protein crystallization, pro-myostatin and 29H4-16 Fab were mixed at a 1:1 molar ratio and incubated at room temperature for 1 h. The pro-myostatin–29H4-16 Fab complex was purified from the constituent components by SEC using HiLoad 16/600 Superdex® 200 pg Column (GE Healthcare) using 30 mm HEPES, 200 mm NaCl, and 5% glycerol (pH 7.4) as eluent buffer.

### Crystallization and data collection

The pro-myostatin–29H4-16 Fab complex was crystallized using sitting drop vapor diffusion by mixing 200 nl of 6.8 mg/ml protein complex with 200 nL of well solution containing 0.1 m Na citrate (pH 5.5), 20% PEG 3350, 15% 2-propanol, and 3% trimethylamine *N*-oxide and equilibrating at room temperature. Crystals were harvested with a cryo-loop and cryoprotected by soaking in well solution with 25% ethylene glycol before plunge-freezing in liquid nitrogen.

Diffraction data were collected from a single crystal at the Advanced Photon Source of Argonne National Laboratory on Industrial Macromolecular Crystallography Association Collaborative Access Team beamline 17-ID-B with a Dectris Pilatus 6 M and a wavelength of 1.00000 Å. The crystal was rotated 180°, and images were collected with 0.5° oscillation and 0.5-s exposure time. The diffraction data were indexed, scaled, and merged using the XIA2 pipeline (CCP4) (62).

### Structure determination

The structure was solved by molecular replacement using PHASER (63) and search models derived from Fabs (PDB codes 5GGU and 5F3H), myostatin (PDB code 3HH2), and pro-TGFβ1 (PDB code 3RJR). Buccaneer (64) was used to automate some of the extension and building/rebuilding of the initial model. Further manual *de novo* building was done to portions of the prodomain that showed generally weak, disconnected density because of the lack of sufficient phasing for this domain. The structure was completed by successive rounds of refinement and manual building in Coot (65), followed by refinement with Refmac5 (49). The subsequent publication of the pro-myostatin precursor (PBD code 5NTU) (16) allowed us to go back and improve many regions of the structure. Portions of the electron density, in regions distant from the myostatin–Fab interface, were weak and discontinuous, creating a challenge for molecular modeling and refinement. This challenge led to a number of bond distance and angle outliers and clashes that were modeled to the best of our ability. This challenge was, in part, motivation for us to corroborate the structure through parallel structural techniques of H/DX-MS and negative-stain EM.

### Binding affinity measurements

The human IgG4 and Fab fragments of 29H4-GL and 29H4-16 were measured for their binding affinities to human pro- and latent myostatin by biolayer interferometry using Octet Red384 (FortéBio). Polystyrene 96-well black half-area plates were utilized for this experiment (Greiner Bio-One). Briefly, pre-equilibrated anti-human IgG Fc capture biosensor tips (FortéBio) were baselined for 1 min in 1× kinetics buffer (FortéBio, catalog no. 18-1105), and antibodies were loaded onto the anti-human IgG Fc capture tips at a concentration of 1 μg/ml for 5 min. An additional 1-min baseline was performed in 1× kinetics buffer followed by a 5-min association step using varying concentrations of human pro- or latent myostatin (0–300 nm). Finally, a 15-min dissociation step was performed.

To determine the affinities of the Fabs to human pro- and latent myostatin, anti-human Fab-CH1 second-generation (FAB2G) biosensor tips (FortéBio) were used. Briefly, pre-equilibrated FAB2G tips were baselined for 5 min in 1× enhanced kinetics buffer (1× kinetics buffer with 2% BSA, 0.5 m NaCl, and 0.09% Tween 20). The Fab was then loaded onto the FAB2G tips at a concentration of 1 μg/ml for 5 min. An additional 1-min baseline was performed in 1× enhanced kinetics buffer followed by a 5-min association step using varying concentrations of human pro- or latent myostatin (0–300 nm). Finally, a 15-min dissociation step was performed.

For analysis and determination of the dissociation constant (*K_D_*) for each antigen/antibody pair, a global fit was performed using all concentrations of antigen for which the association response was greater than 0.1 and the dissociation resulted in a measurable off-rate. For samples where off-rates were not detectable for any of the concentrations, the off-rate and *K_D_* were assigned to be below the limit of detection on the Octet instrument. A 1:1 model was utilized for all curve fitting. The data were analyzed using Data Analysis 11.0 software (FortéBio).

### SEC-MALS

The size and binding stoichiometry of pro- and latent myostatin with SRK-015 were determined by SEC-MALS using 20 mm phosphate buffer and 200 mm NaCl (pH 6.8) as eluent. SEC-MALS was performed using an HPLC (e2695 Separation Module, Waters Corp.) connected to a series of detectors, including a UV-visible multi-angle scattering detector (miniDawn TREOS, Wyatt Technologies) and Optilab T-rREX refractometer (Wyatt Technologies). SEC separation was accomplished using a silica-based SRT SEC-500 column (5 μm, 500 Å, 4.6 × 200 mm, Sepax Technologies). Prior to loading onto the SEC column at 0.3 ml/min, samples of pro- and latent myostatin alone and in complex with SRK-015 were incubated overnight at room temperature. The absolute molar masses were then determined from the light scattering data using ASTRA 6.1 software (Wyatt Technologies).

### ELISA-based myostatin activity assay

The ActRIIb–mouse Fc fusion protein was coated at 2 μg/ml in 1× PBS buffer onto a plate (Costar, catalog no. 3361) and stored overnight at 2 °C-8 °C. The next day, plates were washed and blocked with assay buffer (1% BSA in 1× Tris buffered saline containing, 0.1% Tween20) at room temperature. Anti-myostatin antibodies and Fabs were diluted to 200 μg/ml in 1× PBS and titrated 1:2 in 1× PBS. Human latent myostatin was diluted to 4 μg/ml in activity assay buffer (100 mm HEPES, 1 mm CaCl_2_, 1 μm ZnCl_2_, and 0.01% Brij-35). Antibodies and latent myostatin were added at a 1:1 volume ratio and incubated for 30 min at 37 °C. Conditioned medium containing mTLL2 protease, prepared and validated as described previously (13), was added at a 1:1 volume ratio to samples and incubated for 1 h at 37°C. Myostatin standard (R&D Systems, catalog no. 788-G8-10) was diluted in assay buffer to 200 ng/ml and titrated 1:3 in assay buffer. Samples were diluted 1:20 in assay buffer, and after aspirating the blocking buffer from the assay plate, the samples and myostatin standard were added to the plate and incubated for 90 min at room temperature with agitation (500 rpm). Bound myostatin was detected with biotin goat anti-myostatin (R&D Systems, catalog no. BAF788) diluted to 0.2 μg/ml in assay buffer and incubated for 1 h at room temp with agitation (500 rpm). Bound complex was detected with high-sensitivity streptavidin HRP (Thermo Fisher, catalog no. 21130). After washing, plates were developed by addition of TMB substrate (Surmodics, catalog no. TMBW-1000-01). The reaction was quenched with 1 m phosphoric acid. The absorbance readings at 450 nm were measured using a Synergy H1 microplate reader (BioTek) with a background correction at 630 nm. The limit of detection for this assay was 1 ng/ml based on detection of a recombinant myostatin growth factor standard.

### Hydrogen/deuterium exchange mass spectrometry

H/D exchange experiments were performed using a 1:3 molar ratio of pro- and latent myostatin–29H4-(16/GL) Fab (15 μm each) in sample buffer (20 mm HEPES and 150 mm NaCl (pH 7.5)). A 15 μm pro- or latent myostatin sample was also prepared for the unbound antigen control. The protein samples were incubated at room temperature for 1 h and then introduced into the nanoACQUITY UPLC^TM^ system equipped with H/D exchange technology (Waters Corp.), which was used to perform all subsequent manipulations for the H/D exchange experiments. H/D exchange was initiated by dilution of each sample 10-fold with H/D exchange buffer (20 mm HEPES and 150 mm NaCl, pD 7.5) and the mixture was incubated at predetermined H/D exchange time points (0.17, 1, 10, and 60 min) at 25 °C. At the indicated time point, an aliquot from the exchange reaction was removed and quenched by adding an equal volume of quench buffer (100 mm phosphate, 4 m guanidine HCl, and 0.5 m tris(2-carboxyethyl)phosphine (pH 2.5)) at 3 °C. For the nondeuterated samples, the same procedure was performed in sample buffer. Quenched samples were introduced into the 5 μm ethylene bridged hybrid 2.1 × 30 mm Enzymate^TM^ immobilized pepsin column (Waters Corp.) at 100 μl/min using 0.1% formic acid as eluent at 10 °C and incubated for an additional 9.5 min to allow on-column digestion. Peptide fragments were collected at 0 °C on a C18 VanGuard trap column (1.7 μm × 30 mm, Waters Corp.) for desalting using 0.1% formic acid (in water). The digested peptides were introduced onto the 1.8-μm high strength silica T3 C18 2.1 × 30 mm nanoACQUITY UPLC® column (Waters Corp., Milford, MA) at 40 μl/min (0 °C) using a 10-min gradient from 0.1% formic acid to acetonitrile (7 min, 5%–35%; 1 min, 35%–85%; 2-min hold, 85% acetonitrile). Fragments were subsequently mass-analyzed using the Synapt G2Si electrospray ionization quadrupole time-of-flight mass spectrometer (Waters Corp.). Between each sample run, a clean blank was run by injecting pepsin wash buffer (1.5 m guanidine HCl, 4% acetonitrile, and 0.8% formic acid) into the H/DX-MS system to minimize peptide carryover.

Accurate mass and collision-induced dissociation in data-independent acquisition mode (MS^E^) and ProteinLynx Global Server 3.0 software (Waters Corp.) were used to determine the peptic peptides in the undeuterated protein samples analyzed on the same UPLC-ESI-QTOF system used for H/DX-MS experiments. Peptic peptides generated from the ProteinLynx Global Server were imported into DynamX 3.0 (Waters Corp.) with peptide quality thresholds of MS1 signal intensity of 1000 or higher and a maximum mass error of 1 ppm. Automated results were manually inspected to ensure that the corresponding *m*/*z* and isotopic distributions at various charge states were properly assigned to the appropriate peptic peptide. DynamX 3.0 was used to generate the relative deuterium incorporation plot and H/D exchange heatmap for each peptic peptide. The relative deuterium incorporation of each peptide was determined by subtracting the weight-averaged centroid mass of the isotopic distribution of the undeuterated control sample from that of the weight-averaged centroid mass of the isotopic distribution of deuterium-labeled samples at each labeling time point. All comparisons were performed under identical experimental conditions, negating the need for back-exchange correction in determination of the deuterium incorporation. Thus, H/D exchange levels are reported as relative (51). The fractional relative deuterium uptake was calculated by dividing the relative deuterium uptake of each peptic peptide by its theoretical maximum uptake. All H/DX-MS experiments were performed in duplicate, and a 98% confidence limit for the uncertainty of the mean relative deuterium uptake of ±0.5 Da was calculated as described previously (66). Differences in deuterium uptake between two states that exceed 0.5 Da were considered significant.

### Negative-stain electron microscopy

The samples for negative-stain EM include the antigen–antibody complexes of pro-myostatin–29H4-16 hIgG4 and latent myostatin–SRK-015. Prior to negative-stain EM analysis, formation of an antigen–antibody complex was confirmed by size-exclusion chromatography using Superdex 200 Increase 5/150 GL with 20 mm phosphate buffer and 200 mm NaCl (pH 6.8 as eluent). The samples were combined at a 1:2 molar ratio (antigen:antibody), incubated on ice for 1 h, and diluted 150-fold in 20 mm HEPES and 150 mm NaCl (pH 7.5) prior to imaging. Each sample was loaded onto a carbon-coated, 400-mesh copper grid; the buffer was wicked off, and the sample was immediately stained with uranyl formate. Negative-stain EM imaging was performed using an FEI Eagle 4k × 4k charge coupled device camera. After identifying suitable target areas for imaging at lower magnifications, high-magnification images were acquired at nominal magnifications of ×110,000 (0.10 nm/pixel) and ×67,000 (0.16 nm/pixel) with a nominal underfocus of −1.6 μm to −0.8 μm and electron dose of 25 e^−^/Å. For 2D class averaging, particles were identified at high magnification (×67,000) images prior to alignment and classification. Particles were selected and boxed out, and individual subimages were combined into a stack to be processed using reference-free classification based on the XMIPP (67) processing package. Particles were selected using combined automated (68) and manual picking protocols. An initial round of alignment was done on each sample, and further alignment was performed based on the results from the initial alignments that showed recognizable particles. Based on the observed homogeneity of the samples of antigen–antibody complexes, only two rounds of 2D class averaging were necessary.

## Author contributions

K. B. D., E. T., J. W. J., R. R. F., A. D. C., and G. J. C. conceptualization; K. B. D., E. T., F. C. S., J. W. J., R. R. F., S. C. L., S. B. N., A. D. C., and G. J. C. formal analysis; K. B. D., E. T., F. C. S., J. W. J., A. N., S. C. L., C. J. B., S. B. N., and A. D. C. investigation; K. B. D., E. T., F. C. S., J. W. J., R. R. F., A. N., S. C. L., C. J. B., A. D. C., and G. J. C. methodology; K. B. D., F. C. S., J. W. J., R. R. F., and S. C. L. writing-original draft; K. B. D., S. B. N., and G. J. C. writing-review and editing; C. J. B., S. B. N., A. D. C., and G. J. C. supervision.

## Supplementary Material

Supporting Information

## Footnotes

3 The abbreviations used are:

TGF-β: transforming growth factor beta
H/DX-MS: hydrogen/deuterium exchange mass spectrometry
CDR: complementarity-determining region.
