# Supplementary material for: Structural basis of specific inhibition of extracellular activation of pro- or latent myostatin by the monoclonal antibody SRK-015
Source: J Biol Chem. 2020 Feb 19;295(16):5404–18. doi: 10.1074/jbc.RA119.012293 (PMC7170532; doi:10.1074/jbc.RA119.012293)
Supplement: Supporting Information [file supp_295_16_5404__index.html]

Structural basis for specific inhibition of extracellular activation of pro- or latent myostatin by the monoclonal antibody SRK-015 — SRK-015-mediated inhibition of pro/latent myostatin extracellular activation — Structural basis of specific inhibition of extracellular activation of pro- or latent myostatin by the monoclonal antibody SRK-015 — SRK-015 inhibition of myostatin extracellular activation — Supporting Information 

# Structural basis of specific inhibition of extracellular activation of pro- or latent myostatin by the monoclonal antibody SRK-015

## Supporting Information

- Supporting Information (to be published online) - Supporting Information
