# Supplementary material for: Structural basis of specific inhibition of extracellular activation of pro- or latent myostatin by the monoclonal antibody SRK-015
Source: J Biol Chem. 2020 Feb 19;295(16):5404–18. doi: 10.1074/jbc.RA119.012293 (PMC7170532; doi:10.1074/jbc.RA119.012293)
Supplement: Supporting Information [file supp_RA119.012293_157687_1_supp_477449_q5wsr3.docx]

**Structural basis for specific inhibition of extracellular activation of pro- or latent myostatin by the monoclonal antibody SRK-015**

**Kevin B. Dagbay^1^, Erin Treece^1^, Frederick C. Streich Jr.^1^, Justin W. Jackson^1^, Ryan R. Faucette^1^, Anastasia Nikiforov^1^, Susan C. Lin^1^, Chris J. Boston^1^, Samantha B. Nicholls^1^,Allan D. Capili^1#^ and Gregory J. Carven^1,*^**

From ^1^Scholar Rock Inc., 620 Memorial Drive, 2^nd^ Floor, Cambridge, MA, USA 02139

Running title: *SRK-015-mediated inhibition of pro/latent myostatin extracellular activation*

^#^Present address: Amagma Therapeutics, Waltham, MA,

*To whom correspondence should be addressed: Gregory J. Carven: Scholar Rock Inc., 620 Memorial Drive, 2^nd^ Floor, Cambridge, MA, USA 02139; [gcarven@scholarrock.com](mailto:gcarven@scholarrock.com); Tel. (857) 259-3860; Fax. (866) 493-4935

**Supporting Information**

**Table S1**. Crystallographic data collection, processing, and refinement statistics.

**Figure S1**. Coverage map of the peptic peptides of pro-myostatin and latent myostatin identified from H/DX-MS.

**Figure S2**. H/DX heat map of the relative deuterium incorporation of pro-myostatin and latent myostatin between its unbound and Fab-bound (SRK-015 Fab or 29H4-16 Fab) states.

**Figure S3**. Relative deuterium uptake plots of pro-myostatin between its unbound and Fab-bound (SRK-015 Fab or 29H4-16 Fab) states over the time course of the H/D exchange experiment.

**Figure S4.** Relative deuterium uptake plots of latent myostatin between its unbound and Fab-bound (SRK-015 Fab or 29H4-16 Fab) states over the time course of the H/D exchange experiment.

**Figure S5.** Negative stain EM class averages of (A) pro-myostatin:29H4-16 hIgG4 and (B) latent myostatin:SRK-015 after the second round of alignments.

**Table S1.** **Crystallographic data collection, processing, and refinement statistics.**

| **Description** | **ProMyostatin:29H4-16 Fab** |
| --- | --- |
| PDB Code | 6UMX |
| ***Data Collection*** | |
| Synchrotron/Beamline | IMCA-CAT; 17-ID-B |
| Wavelength (å) | 1.00000 |
| *Data Processing* | |
| Space group | P2_1_2_1_2_1_ |
| Unit cell (a, b, c) [å] | 59.62, 110.01, 293.27 |
| α, β, γ [°] | 90.0, 90.0, 90.0 |
| Resolution range (å) | 40.43-2.79 |
| No. of protomers in ASU | 2 Pro-Myostatin, 2 29H4-16 Fab |
| No of total/unique reflections | 253235/49196 |
| Multiplicity/Redundancy | 5.1 (5.3) |
| *R_sym_* | 0.035 (0.645) |
| CC_1/2_ | 0.99 (0.545) |
| *I*/*σI* | 14.1 (1.2) |
| Completeness (%) | 99.8 (99.9) |
| Phasing (MR), PDB ID | 5GGU, 5F3H, 3HH2, 3RJR, 5NTU |
| ***Refinement*** | |
| *R_work_/R_free_* | 0.217/0.264 |
| No. of unique/free reflections used | 49196/2372 |
| No. of atoms/Average B-factors |  |
| Protein | 10,849 / 97.2 |
| Ligand/ion | 6 / 85.07 |
| Water | 2 / 65.18 |
| Protein Residues | 1427 |
| Mean/Wilson B-factor | 97.0/81.1 |
| RMS deviations |  |
| Bond lengths (å) | 0.019 |
| Bond angles (º) | 2.072 |
| Ramachandran statistics | |
| % Favored | 94 |
| % Allowed | 5 |
| % Outliers | 1 |

Values in parentheses refer to the highest resolution shell.


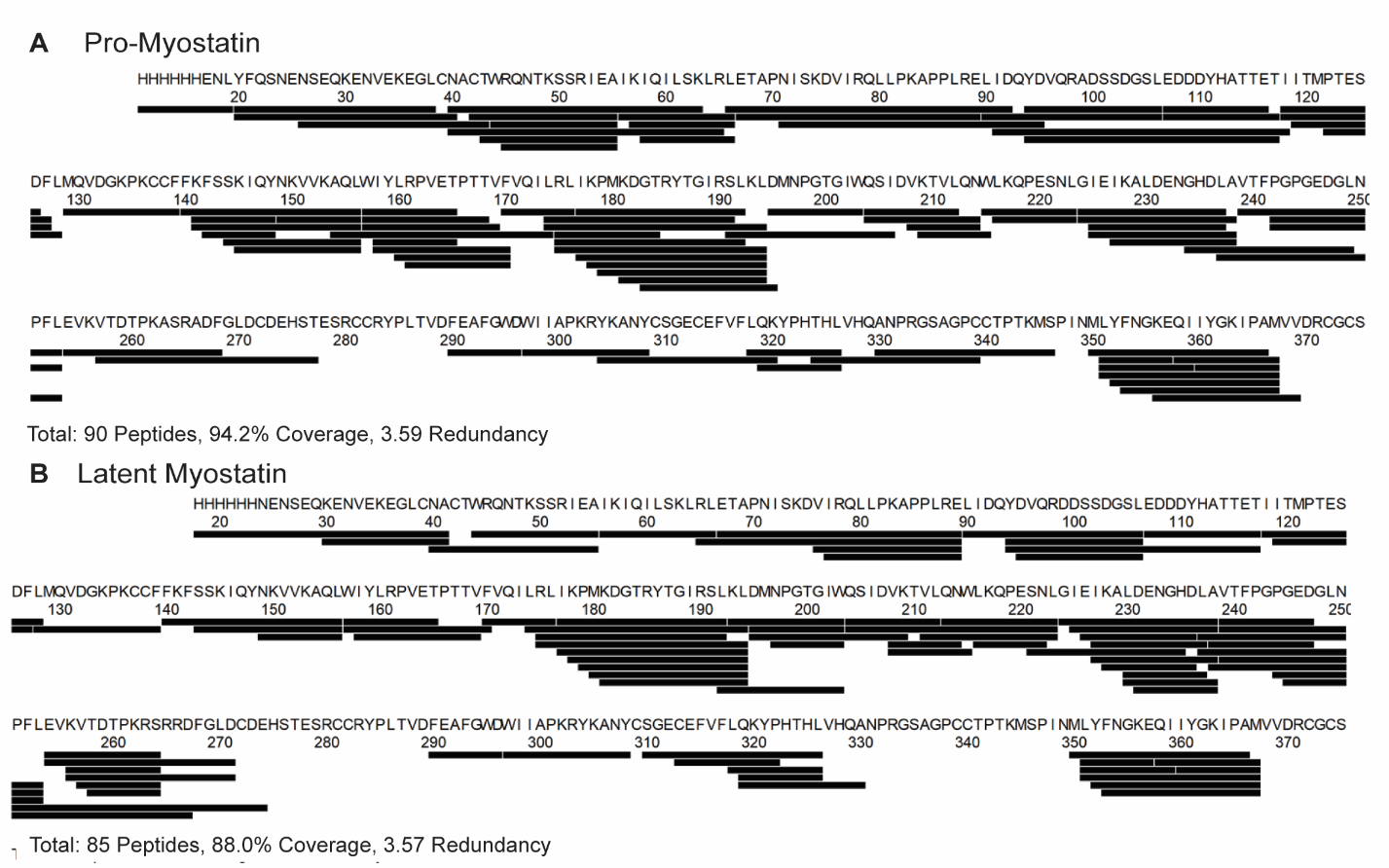


**Figure S1.** Coverage map of the peptic peptides of (A) pro-myostatin and (B) latent myostatin identified from H/DX-MS. The linear amino acid sequence coverage was 94.2% and 88% for pro- myostatin and latent myostatin, respectively. The average redundancy was found to be 3.59 and 3.57 for pro-myostatin and latent myostatin, respectively.

**
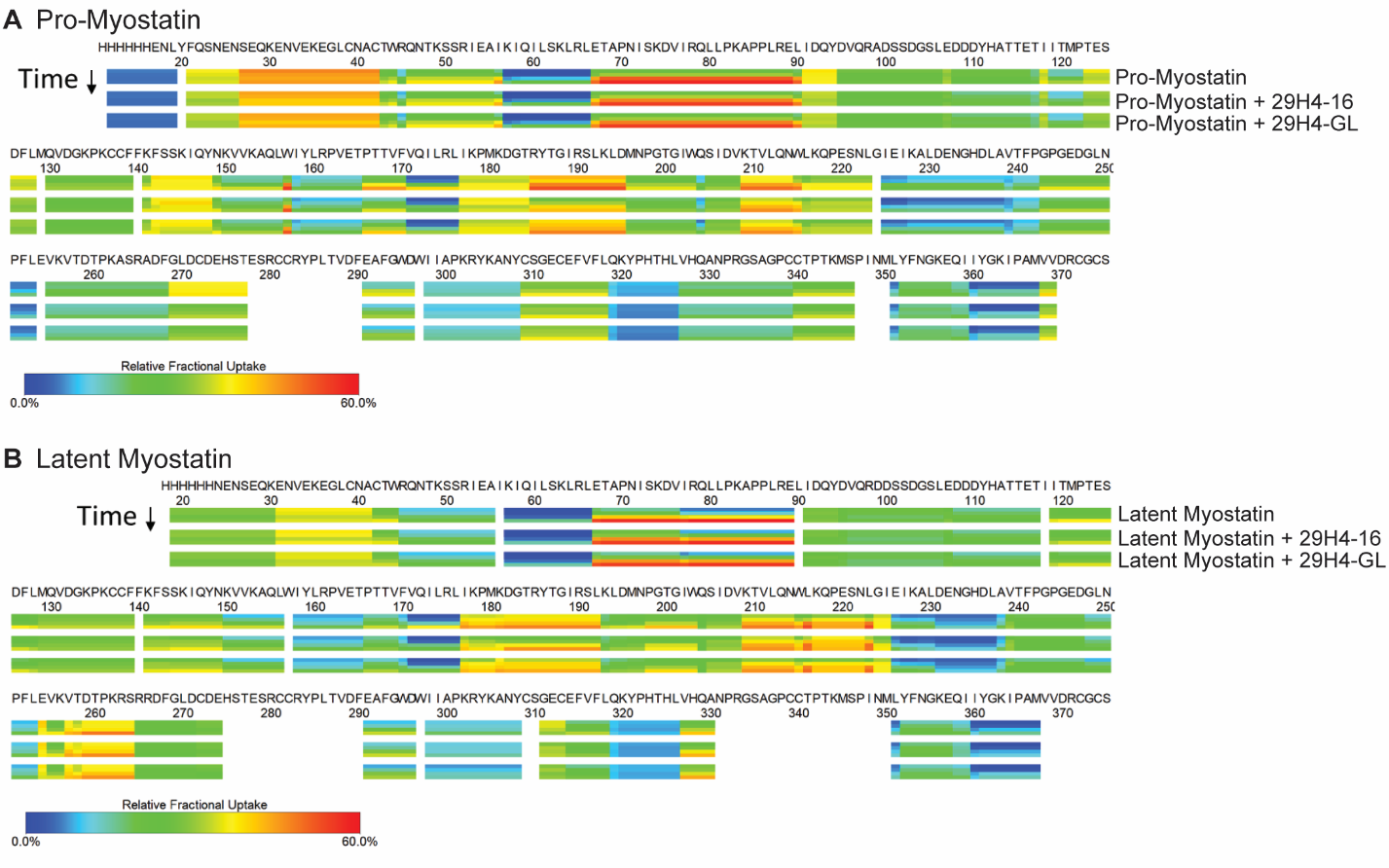
**

**Figure S2.** H/DX heat map of the relative deuterium incorporation of (A) pro-myostatin and (B) latent myostatin between its unbound and Fab-bound (SRK-015 Fab or 29H4-16 Fab) states. For each peptic peptide, the percentage relative deuterium level for each H/DX labeling time (minutes: 0.17, 1, 10, and 60) was mapped onto the corresponding linear sequence of pro- and latent myostatin. The percent relative deuterium incorporation was calculated by dividing the observed deuterium uptake by the theoretical maximum deuterium uptake for each peptide. Blank white spaces in the H/DX heat map represent peptide regions with no coverage, thus H/DX data were not reported.


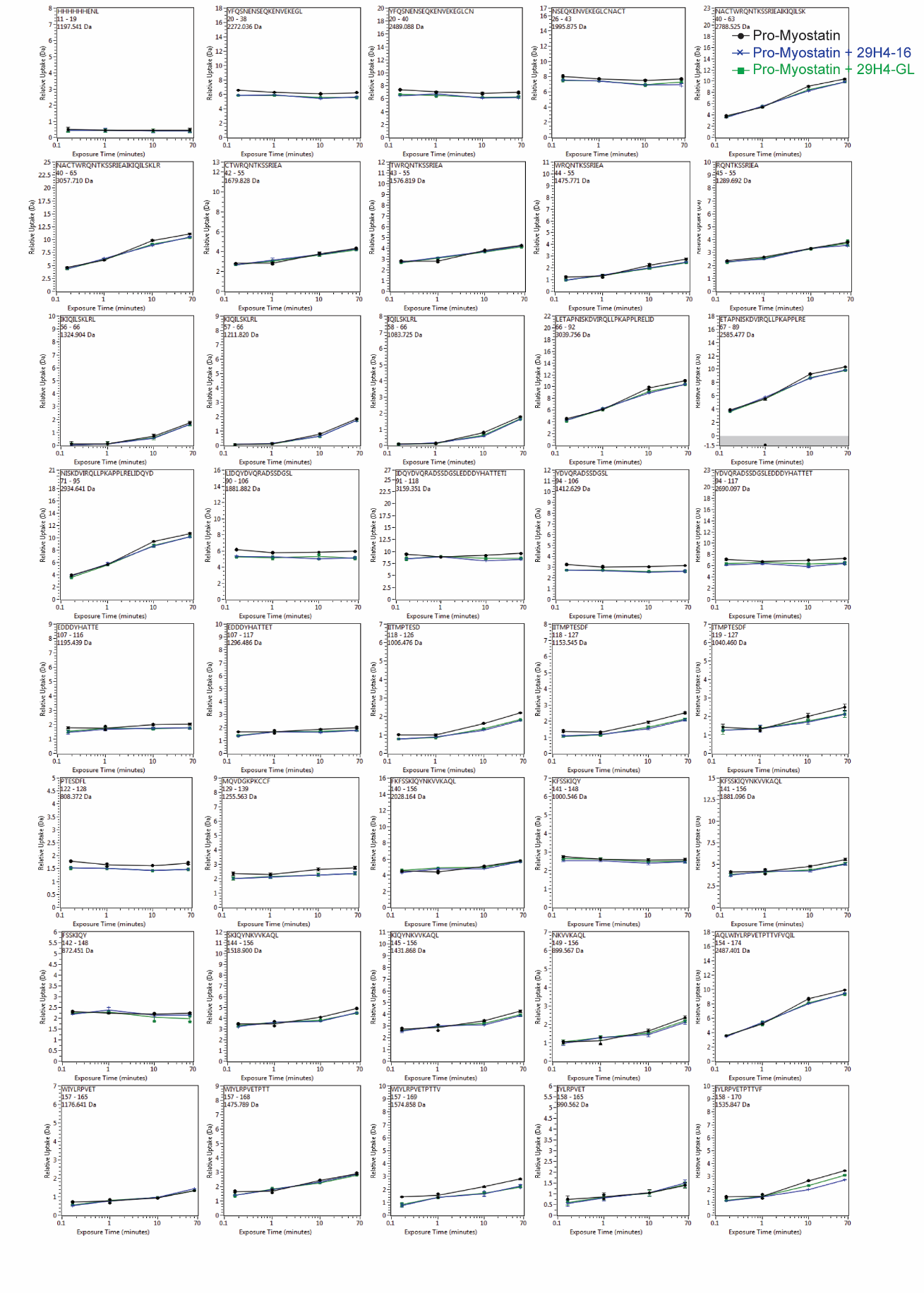


**
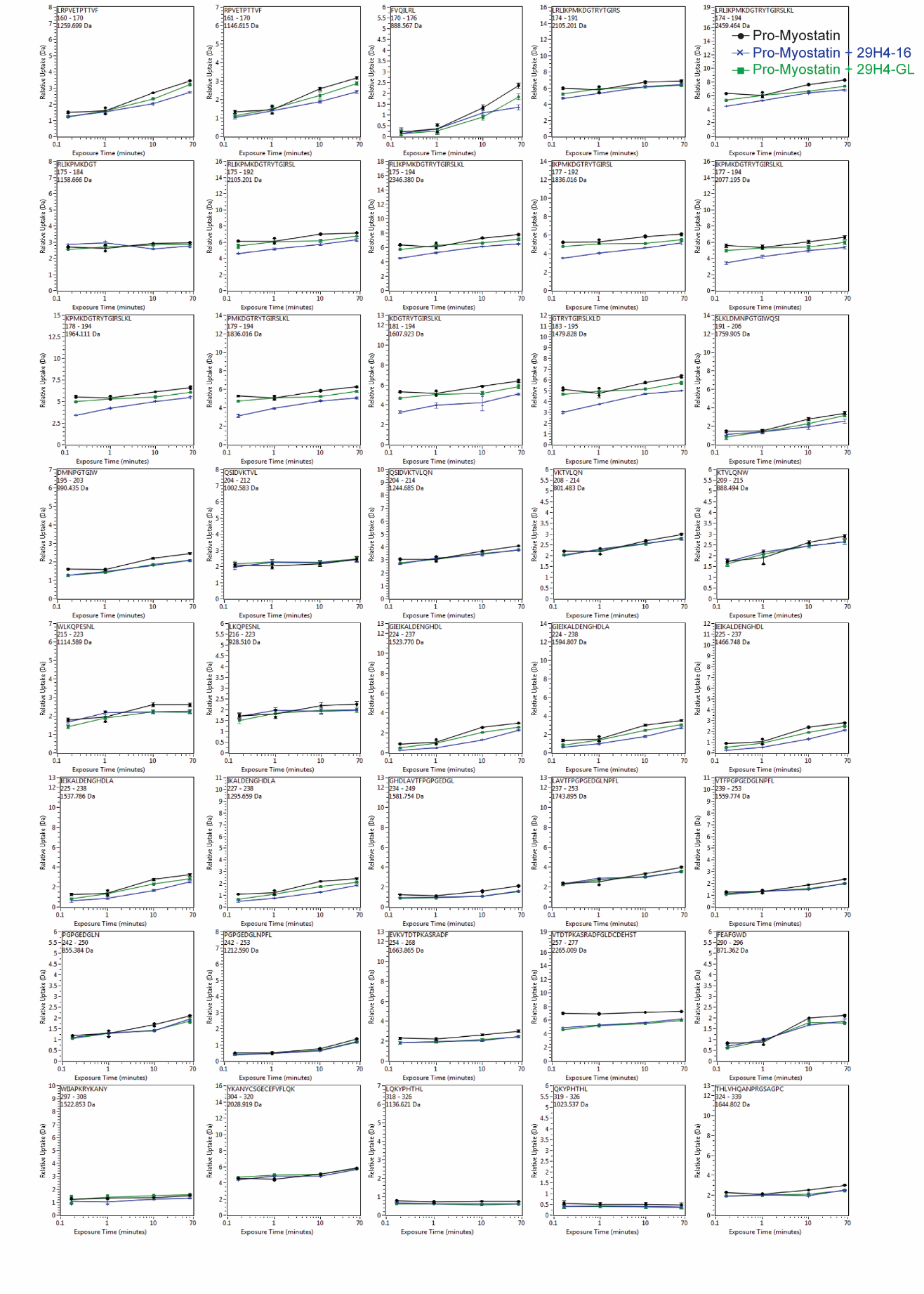
**

**
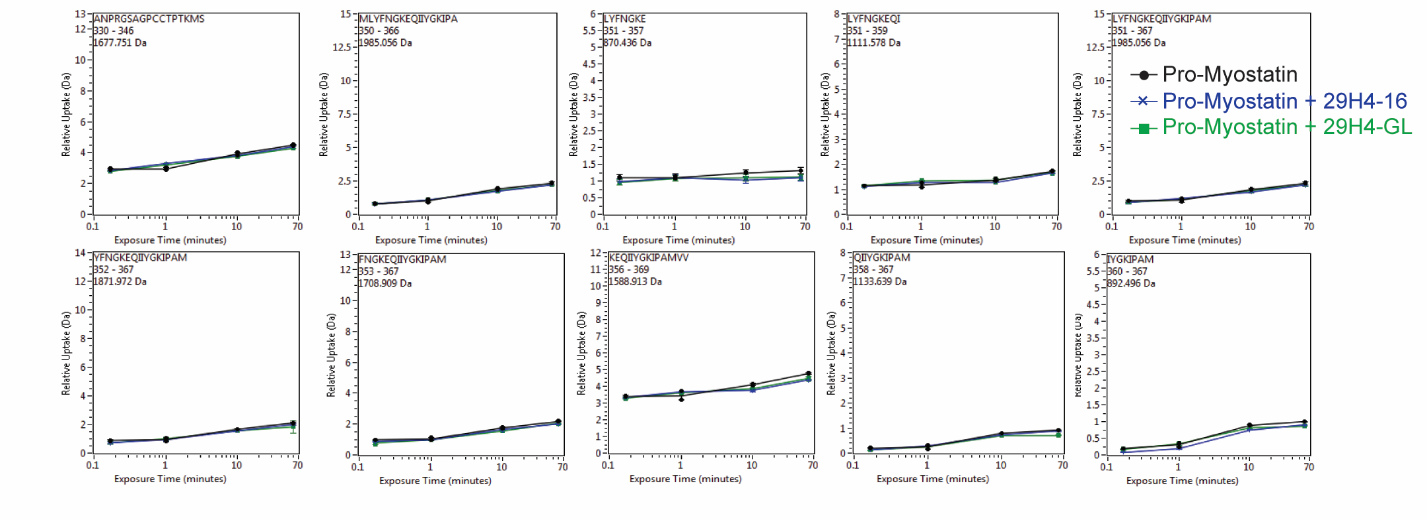
**

**Figure S3.** Relative deuterium uptake plots of pro-myostatin between its unbound and Fab-bound (SRK-015 Fab or 29H4-16 Fab) states over the time course of the H/D exchange experiment. The covered residues for each peptic peptide of pro-myostatin are indicated. *Error*, SD of duplicate H/DX-MS measurement done on two separate days. Figure S3 includes all uptake plots, including the representative deuterium incorporation plots of key peptic fragments which are included in Figure 3E in the main text.

**
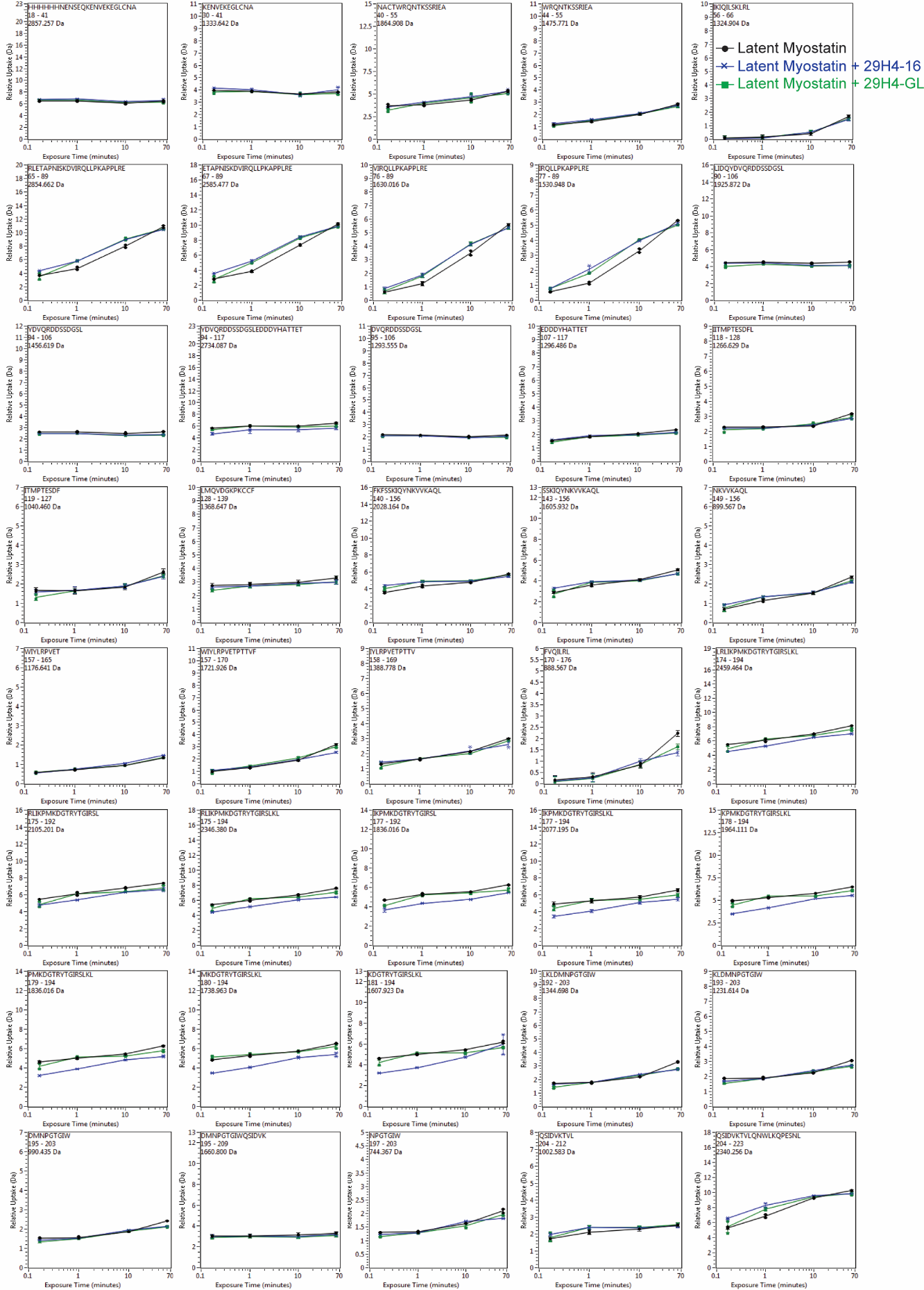
**

**
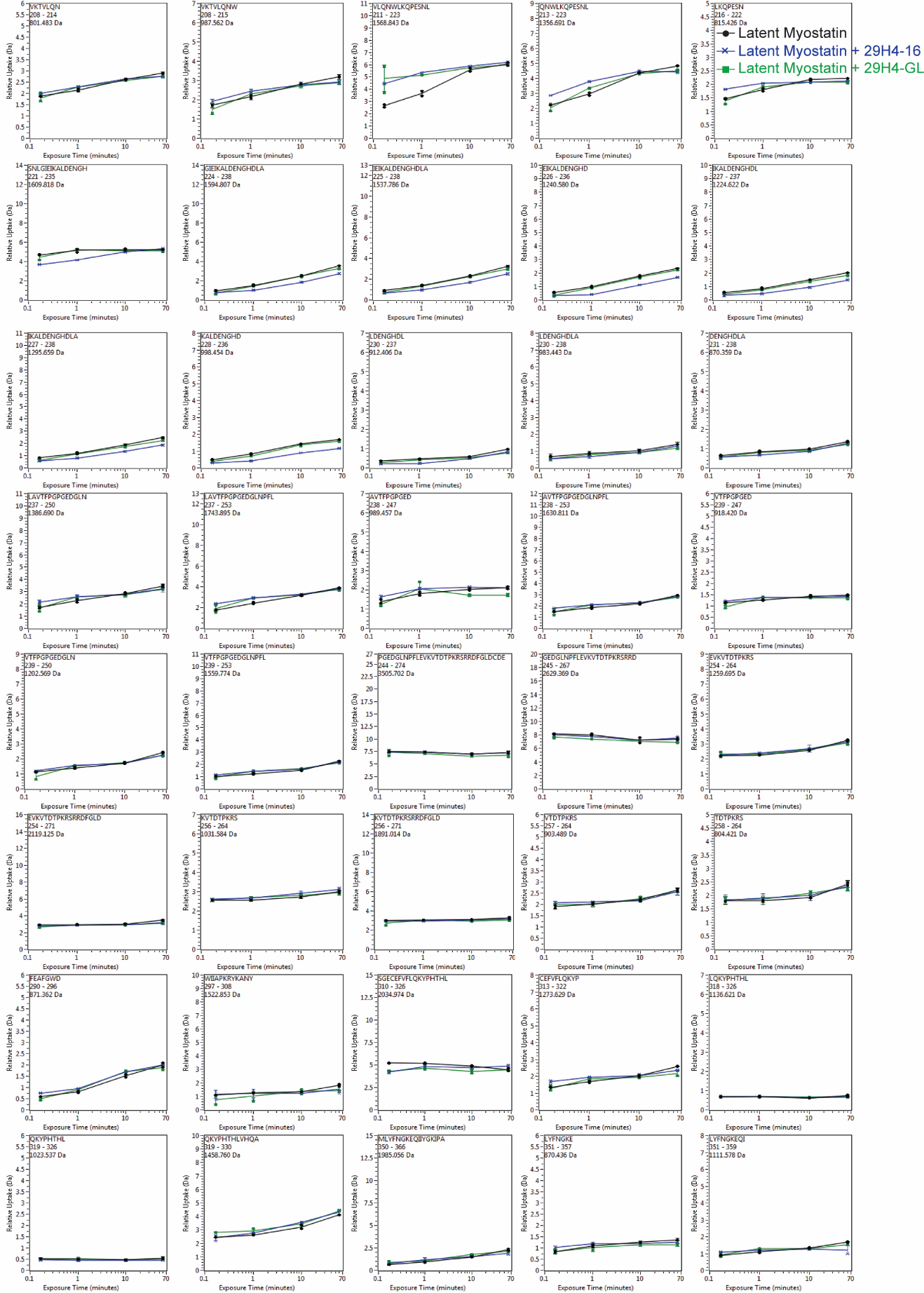
**

**
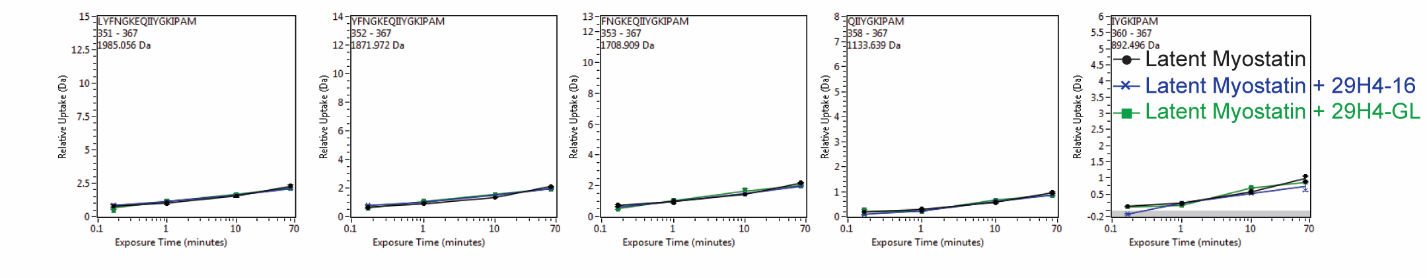
**

**Figure S4.** Relative deuterium uptake plots of latent myostatin between its unbound and Fab-bound (SRK-015 Fab or 29H4-16 Fab) states over the time course of the H/D exchange experiment. The covered residues for each peptic peptide of latent myostatin are indicated. *Error*, SD of duplicate H/DX-MS measurement done on two separate days. Figure S4 includes all uptake plots, including the representative deuterium incorporation plots of key peptic fragments which are included in Figure 3F in the main text.

**
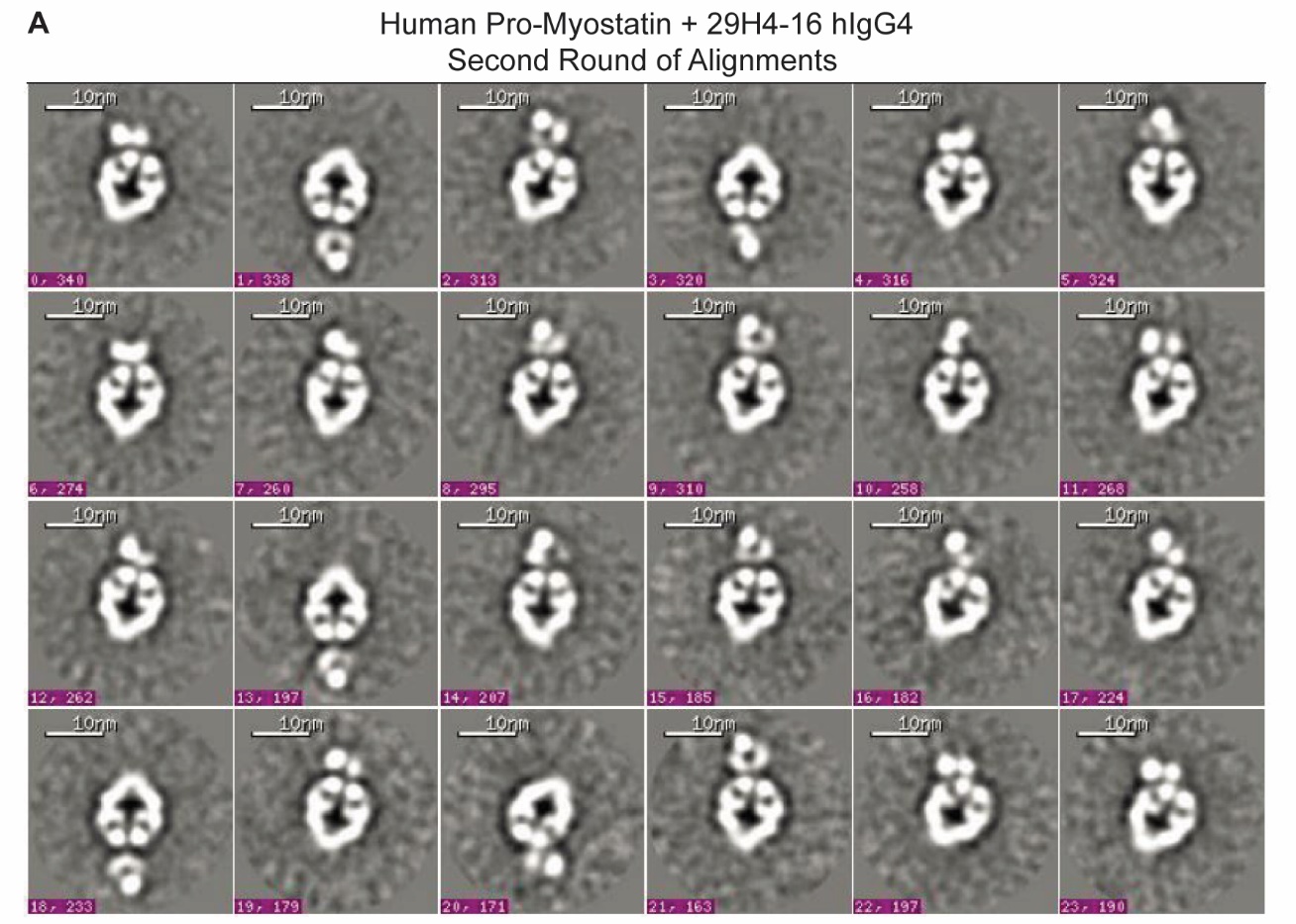
**

**
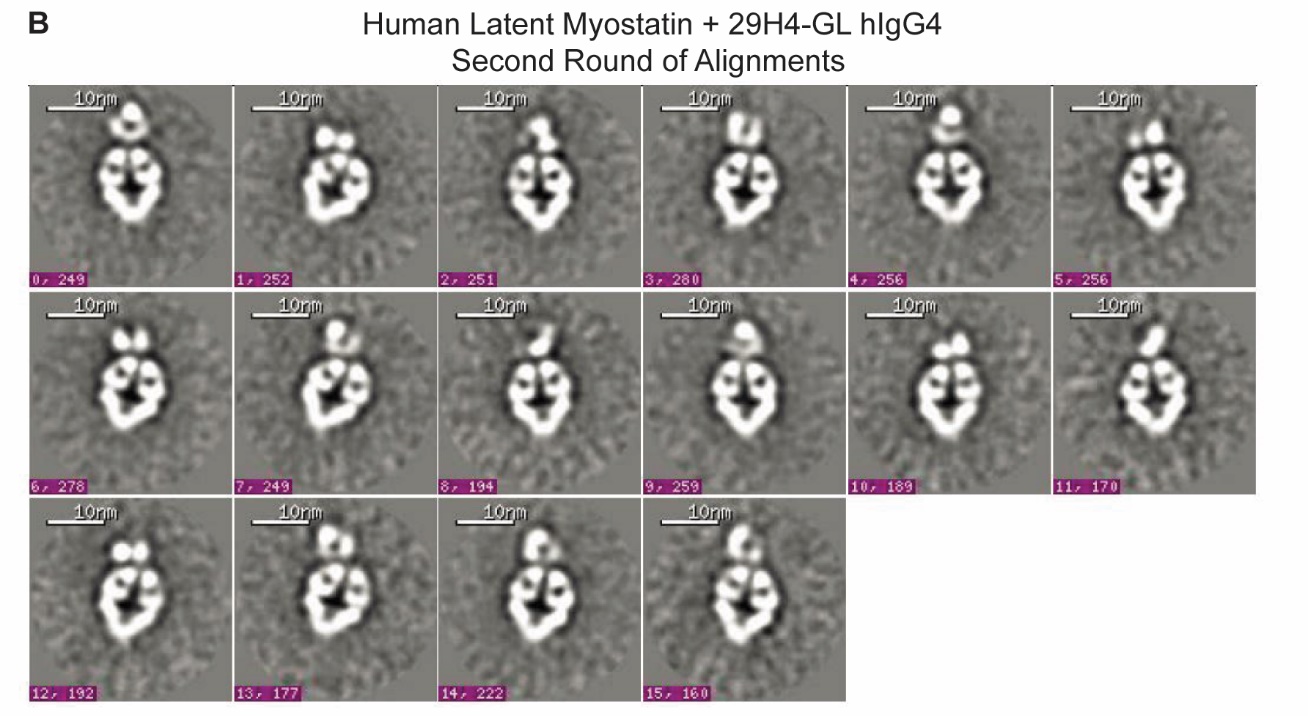
**

**Figure S5.** Negative stain EM class averages of (A) pro-myostatin:29H4-16 hIgG4 and (B) latent myostatin:SRK-015 after the second round of alignments. The first number at the bottom of each particle states the class number. The second number states the number of particles contributing to that class. For ease of comparison, Figure S5 includes the class averages from Figure 4 (B-E) and depicts a broader representation of the class averages for (A) pro-myostatin:29H4-16 hIgG4 and (B) latent myostatin:SRK-015.
